# Pediatric skull base tumors: Clinical features and surgical outcomes; a single center retrospective study with a review of literature

**DOI:** 10.1016/j.bas.2024.104136

**Published:** 2024-11-13

**Authors:** M. Grutza, P. Lenga, M. Issa, A. Seitz, F. Sahm, T. Milde, A.W. Unterberg, S.M. Krieg, A. El Damaty

**Affiliations:** a Department of Neurosurgery, Heidelberg University Hospital, Heidelberg, Germany; b Medical Faculty of Heidelberg University, Heidelberg, Germany; c Department of Neuroradiology, Heidelberg University Hospital, Heidelberg, Germany; d Department of Neuropathology, Institute of Pathology, Heidelberg University Hospital, Heidelberg, Germany; e Hopp Children's Cancer Center Heidelberg (KiTZ), Heidelberg, Germany; f Clinical Cooperation Unit Pediatric Oncology, German Cancer Research Center (DKFZ) and German Consortium for Translational Cancer Research (DKTK), Heidelberg, Germany; g National Center for Tumor Diseases (NCT), Heidelberg, Germany; h University Hospital Jena, Department of Pediatrics and Adolescent Medicine, Friedrich Schiller University Jena, Jena, Germany

**Keywords:** Skull base tumors, Pediatric population, Pediatric neurooncology

## Abstract

**Objectives:**

Pediatric intracranial tumors, particularly at the skull base, are rare and present unique challenges to pediatric neurosurgeons and oncologists, owing to their complex anatomy and diverse histopathology. Robust evidence is still marginal concerning their clinical and surgical courses. Our aim is to describe our experience regarding surgical approaches, with special focus on surgical features, postoperative outcomes, adverse events as well as adjuvant therapeutic concepts.

**Methods:**

Patients aged <18 years undergoing skull base surgery between 2017 and 2023 at our institution were retrospectively enrolled. Patient demographics, tumor characteristics, surgical approach, pre -and postoperative clinical status and adjuvant therapy as well as overall and progression free survival were assessed.

**Results:**

Twelve children aged 6.1 ± 4.1 years were analyzed. There was a predominance of the female gender (7/12, 58.4%). Mean tumor diameter was 4.0 ± 2.9 cm. In three children the tumor was located suprasellar, temporobasal in one, adjacent to the cerebellar pontine angle in 4, clival in 3 and petroclival in 2 children. A subfrontal approach was performed in two patients, a subtemporal approach in one, a retrosigmoidal approach in 5 and in one patient two-staged approach; retrosigmoidal and later in a second operation pterional approach was conducted. One clival tumor was removed via an endonasal endoscopic approach and in another case via a transoral endoscopic approach. Gross total resection (GTR) and near total resection were achieved in 7 patients (58.3%). Tumor types included meningioma, clivus-chordoma, epidermoid cyst, anaplastic ependymoma, Ewing's sarcoma and Atypical Teratoid Rhabdoid Tumor (ATRT) as well as embryonal tumor with multilayered rosettes (ETMR). 4 patients (33.3%) died of disease due to tumor progression in average after 15 months. Hydrocephalus developed in two patients; a ventriculoperitoneal shunt was inserted in one patient, while an endoscopic third ventriculostomy (ETV) was performed in the other. Moreover, one child suffered from a residual neurological deficit at last follow-up evaluation. Adjuvant therapy protocols were applied in six patients (50.0%).

**Conclusion:**

Skull base tumors in children present a therapeutic challenge due to their rarity and unique pathological composition and can lead to considerable morbidity and mortality. An interdisciplinary approach involving neurosurgeons, pediatric oncologists and radiotherapists is mandatory to guarantee the best clinical course.

## Highlights

• Our study explores the complexities of pediatric skull base tumors, highlighting key strategies that enhance survival rates and reduce complications through surgical precision and coordinated interdisciplinary care.
• This retrospective analysis provides an in-depth examination of surgical techniques for rare pediatric skull base tumors, offering valuable insights into effective treatment strategies.
• The study advocates for groundbreaking, collaborative approaches, setting a new benchmark for comprehensive care in pediatric oncology.

## 1 Introduction

Intracranial tumors are rare in the pediatric population, especially those located at the skull base.

The types of pediatric brain tumors (PBTs) and their incidence differ based on a variety of factors such as age, sex, geography, race, and ethnicity. In the United States, for example, the incidence of all primary brain and other CNS tumors in children and adolescents under 20 years old was reported to be 6.06 per 100,000 children from 2012 to 2016. Of these, approximately 58% were malignant (Adel et al., 2021).

The epidemiological landscape of intracranial tumors showcases a distinct variance between adult and pediatric populations, with meningiomas serving as a prime example. Although they stand as the predominant primary intracranial neoplasm in adults, their occurrence in children is comparatively rare (Navarria et al., 2015; Stanuszek et al., 2014). This rarity is accentuated within the pediatric skull base region, where such tumors are even less frequent—a phenomenon highlighted in recent studies (Burkhardt et al., 2013), (Wang et al., 2015).

Pediatric neuro-oncology also confronts the predominance of certain malignancies that are virtually exclusive to early childhood. Atypical Teratoid Rhabdoid Tumors (ATRTs) and Embryonal Tumors with Multilayered Rosettes (ETMRs) exemplify this category. ATRTs account for a small fraction of pediatric CNS tumors but are most commonly diagnosed in the very young, under the age of three (Nesvick et al., 2020). Similarly, ETMRs, while rare and aggressive, primarily affect infants. Despite advancements in diagnostic techniques, such as the discovery of C19MC amplification and DNA-methylation-based classification, comprehensive epidemiological data on ETMRs are lacking, mirroring the broader challenge of accurately characterizing these tumors (Ostrom et al., 2019).

Outcomes for skull base surgery are well established in adults, but data on its use in children are scarce. Pediatric skull base surgery presents unique challenges, including smaller anatomical structures, unerupted teeth, a developing skull base with shifting landmarks, and potential long-term effects on craniofacial growth (Tsai et al., 2002; Beniwal et al., 2024). Due to the rarity of these lesions in children, there is limited agreement on treatment strategies, the extent of resection required, and expected outcomes. Although adult studies increasingly focus on quality of life and functional outcomes after surgery, such research in pediatric patients remains limited.

This discussion acknowledges the overlapping concerns and treatment approaches between different age groups while also recognizing the need for pediatric-specific management strategies due to the unique clinical features of these tumors. The paucity of data, particularly regarding skull base tumors in children, emphasizes the importance of compiling and sharing clinical experiences. In this regard, we aim to scrutinize etiological factors, asses associated morbidity and mortality rates, present surgical approaches, evaluate long-term clinical outcomes and subsequent therapeutic strategies in children operated on skull base tumors.

## 2 Methods

### 2.1 Study design, inclusion and exclusion criteria

This is a retrospective, single center case series study. This study was approved by the local ethics committee (approval number S307/2023) and conducted in accordance with the Declaration of Helsinki. Informed consent from patients' parents or guardians were collected prior to study inclusion. Inclusion criteria were age <18 years, imaging-based and histologically confirmed diagnosis of a skull base tumor, surgical resection at the Heidelberg University Hospital, and a minimum follow-up period of 6 months. Clinical and imaging data were retrospectively collected for the period between January 2017 and December 2023 from our institution's database. Patients with incomplete clinical or radiographic data were excluded from the study. To reduce the expected heterogeneity of a pediatric series of skull base tumors, optic glioma, craniopharyngioma, typical pituitary adenoma and fibrous dysplasia were excluded from our cohort.

### 2.2 Patient demographics, clinical and radiological data

Clinical and demographic parameters, including follow-up duration, tumor entity, and tumor recurrence, were assessed. Data regarding clinical presentation, postoperative course, and patient disposition were recorded. Surgical parameters, such as the type of surgical approach and surgery-related complications, were analyzed. Extent of tumor resection was based on postoperative imaging. Patients were stratified according to the extent of tumor resection (biopsy (<10%), partial resection (PR; 10–50%), subtotal resection (STR; 51–90%), near-total resection (NTR; >90%), and gross total resection (GTR) as determined by surgical observation and postoperative magnetic resonance imaging (Wisoff et al., 1998). Radiographic measurements, including tumor diameter were recorded. Patients were recorded in study registry according to their diagnoses and were followed up via imaging according to the recommended protocol. If tumor recurrence was observed, the protocol was adjusted accordingly. For malignant tumor entities adjuvant therapy (radiotherapy ± chemotherapy) was also applied according to treatment protocol of each entity.

### 2.3 Outcome assessments

Functional outcome was assessed comparing preoperative to the last available follow-up using the Lansky play-performance scale and the Late Effects Severity Score (LESS) for pediatric patients. The LESS was performed for each patient (Table 4) to document neurocognitive outcomes.

### 2.4 Literature review

A literature search was conducted in PubMed, covering publications up to December 2023. The search was focused on “pediatric skull base tumors,” specifically targeting articles in English that concentrated on the pediatric demographic. Inclusion criteria were set to encompass studies reporting on groups of 10 or more pediatric patients (younger than 18 years) who had undergone surgical procedures. Exclusions were made for studies that provided insufficient data or that were narrowly focused on a specific type of tumor or a particular anatomical location. The analysis was confined to data from patients deemed to have valid information, as not all studies offered complete datasets.

### 2.5 Statistical analysis

Descriptive statistics, including numbers, percentages, mean, and standard deviation, were used to present the data. Statistical tests, such as independent t-tests and chi-squared tests, were employed for groupwise comparisons of baseline characteristics, surgical approach, perioperative and postoperative complications. The statistical significance level was set at p ≤ 0.05. All analyses were conducted using SPSS 29 (IBM-Corp, Armonk, NY, USA).

## 3 Results

### 3.1 Patient demographics and baseline characteristics

In total, 12 children <18 years who underwent multidisciplinary skull base approaches for surgical resection of tumors during the study period were included. The overall mean age was 6.1 ± 4.1 years ranging from 3 to 17 years. Among the patient population, 7 (58.4 %) patients were female, and 5 (41.6%) were male. The median follow-up duration was 20 months (range 6–60 months). Of the patients studied, 25% (3/12) had benign lesions, while the remaining 75% (9/12) harbored a malignant pathology. The mean tumor diameter was 4 ± 1.9 cm, ranging from 1.1 to 6.2 cm. There were 4 deaths in the series due to disseminated malignancy and progression. The median length of stay in the hospital was 21 days (range 6–44 days). The general findings are described in Table 3.

### 3.2 Surgical characteristics

Tumor types included meningioma, clivus-chordoma, epidermoid cyst, anaplastic ependymoma, Ewing's sarcoma, Atypical Teratoid Rhabdoid Tumor (ATRT), embryonal tumor with multilayered rosettes (ETMR) as well as medulloblastoma. In two children the tumor was located suprasellar, temporobasal in one, four were adjacent to the cerebellar pontine angle, three clival tumors and two petroclival masses. A subfrontal approach was performed in two patients, a subtemporal approach in one patient, a retrosigmoidal approach in five patients and in one patient we adopted two-stages approach; retrosigmoidal and later pterional approach. One clival tumor was resected via an endonasal endoscopic approach, in one patient via a transoral approach and in one patient via a retrosigmoidal approach. GTR, NTR, STR and biopsy was achieved in 3 patients (25.0%), 4 (33.3%) patients, 4 (33.3%) patients and 1 (8.4%) patient respectively. Biopsy only was done in one case of a five year old child with a small incidental clival mass which was radiologically suspecting Langerhans Cell Histiocytosis with an inconclusive intraoperative frozen section. The integrated diagnosis showed then chordoma for which the patient received adjuvant proton therapy. The reasons for not performing GTR in all other cases included the avoidance of potential injury to anatomical structures. In these cases, the tumor had already breached the arachnoid planes of the nearby anatomical structure, making it difficult to establish a clear boundary between the tumor and the given/respective structure. In these cases, complete resection would have been only possible with high risk of disruption of the integrity and/or function of these structures. Additionally, the tumor's characteristics played a significant role. Resection of the tumor was considered incomplete if it possessed a firm and hard consistency, necessitating that residual tissue to be left near sensitive structures, such as the optic system or brainstem, to avoid potential damage. The surgical characteristics of each patient is summarized in Table 1.

**Table 1:** Overall patient characteristics.

Case No.	Age (yrs),Sex	Pathology and moleculargenetic report	Localization	Diameter (cm)	Approach	Extent of resect	Tumor remnants	LOS (days)	Radio	Chemo	Recurrence/Progression (month)
1	3, F	Ewing-Sarcoma	Left temporobasal	6.2	Lt subtemporal	GTR	no	30	yes	yes	no
		CD99 strongly positive;									
		EWSR1-FLI1 Fusion detected;									
2	2, M	Anaplastic Ependymoma (WHO grade 3)	Posterior fossa with transtentorial growth	5.5	Suboccipital	NTR	Brainstem	33	yes	yes	Yes, 5
		INI1 Expression retained;									
		Lin28 Tumor Cells negative; p53 Accumulation in tumor cell nuclei									
3	2, F	Metastasis of neuroblastoma (adrenal gland)	Lt CPA	2	Lt retrosigmoidal	STR	-N. vestibulocochleare -N. facialis	17	Yes	yes	Yes, 8
		MYCN negative;									
		1p Deletion negative;									
		VMS/HVS positive;									
		MIBG positive									
4	17, F	Dermoid cyst	suprasellar	1.1	Rt pterional (subfrontal)	GTR	No	10	no	no	no
		N.d.									
5	8, M	Dermoid cyst	suprasellar	2.1	Rt pterional (subfrontal), endoscopic assisted	GTR	No	6	no	no	no
		N.d.									
6	9, F	Meningioma WHO Grade 1	Rt Petroclival (infra -and supratentorial growth)	4.4	Rt retrosigmoidal, Rt pterional	STR	Brainstem, A. basilaris, A. cerebri media (M1), A, carotis interna	44	yes	no	no
		Detection of a YAP1-LMO1 fusion									
7	5, M	Chordoma N.d.	Clivus	6.5	Transoral (extern) 10/2017 Transnasal (extern) 12/2017 Rt retrosigmoidal	STR	Clivus Rt N. hypoglossus	20	Yes	No	no
8	5, F	Chordoma N.d.	Clivus	6.2	Rt sigmoidal (extern), Transnasal endoscopic assisted approach	STR	Medulla oblongata	7	yes	no	no
9	5, M	Chordoma N.d.	Clivus	NA	Transoral	Biopsy		7	yes	No	no
10	6, M	Atypic teratoidic/. rhabdoidic tumor (AT/RT) WHO Grade IV	Rt CPA	2	Rt retrosigmoidal	NTR	Meatus acusticus internus, PICA	42	yes	yes	yes, 24
		homozygous deletion of the SMARCB1 gene									
11	3, F	Embryonal tumor with Multilayered Rosettes (ETMR)	Rt petroclival	3.5	Rt retrosigmoidal	NTR	Brainstem, A. basilaris	19	yes	yes	Yes, 5
		IDH1 R132H: negative nuclear;									
		ATRX expression retained;									
		BRAF V600E negative;									
		H3 K27M negative;									
		LIN28A partially positive in tumor cells;									
		MGMT promoter unmethylated									
12	8, F	Medulloblastoma	RT CPA	4.1	Rt retrosigmoidal	NTR	Brainstem	19	yes	yes	Yes, 4
		non-WNT/non-SHH;									
		group 4;									
		subclass VII;									
		LIN28 negative;									
		ATRX expression retained;									
		INI1 expression retained;									
		IDH1 R132H negative									

### 3.3 Functional outcome, postoperative morbidity and mortality

In our cohort, the preoperative mean Lansky score was 80, with a range from 50 to 100. One patient (8.3%) developed a new postoperative neurological deficit, which improved from House-Brackmann Grade III postoperative to Grade II after 6 weeks then 1 year later was completely normal HB Grade I. Hydrocephalus occurred in two patients; one received a ventriculoperitoneal shunt, while the other underwent an endoscopic third ventriculostomy (ETV). 4 patients (33.3%) died of disease due to tumor progression in average after 15 months with a range from 6 to 29 months.

The postoperative outcomes were as follows: The mean Lansky score increased to 88.7, with a range from 50 to 100. The average Late Effects Severity Score (LESS) was 1, ranging from 0 to 1. Patients with benign tumors had an average Lansky score of 100 and a LESS score of 0, while those with malignant tumors had a mean Lansky score of 81.6 and a LESS score of 1.5. These findings are summarized in Table 2.

**Table 2:** Functional outcome.

Case No.	Preoperative Lansky-Score	Preoperative Symptoms	Postoperative morbidity and mortality	Postoperative LESS	Postoperative Lansky-Score
1	80	Left-sided facial palsy with corneal opacity	Remitting left-sided abducens palsy	0	100
2	50	Torticollis, gait instability, apathy, vomiting	Implantation of VP-Shunt by hydrocephalus occlusus, death after 12 month	3	50
3	80	Left-sided facial palsy	VP-Shunt because of hydrocephalus	NA	NA
			Death after 12 month		
4	100	No	No	0	100
5	100	No	No	0	100
6	60	Headache, gait instability, right-sided abducens palsy, right-sided facial palsy	No	1	100
7	70	Paresis of N. Hypoglossus, N. Glossopharyngeus, N. reccurens because of previous operation	No	2	70
8	90	Since first operation right abducens paresis	No	3	90
9	90	No	No	0	100
10	80	Right-sided facial palsy House & Brackmann grade IV, right-sided hearing loss	Death after 29 month	NA	NA
11	80	Mild right-sided facial palsy	Death after 6 month	NA	NA
12	80	Headache, gait instability	Postoperative ETV because of hydrocephalus	0	80

**Table 3:** Baseline characteristics.

Number of Patients	12
Age, years (mean, SD)	6.1 (4.1)
Sex (n, %)	
Male	5 (41.6)
Female	7 (58.4)
Longest follow up in month (mean, SD)	19.6 (16.9)
Tumor diameter (mean, SD, cm)	4.0 (1.9)
Extension of resection (n, %)	
Biopsy	1 (8.4)
STR	4 (33.3)
NTR	4 (33.3)
GTR	3 (25.0)
Adjuvant therapy (n, %)	
Chemotherapy	6 (50.0)
Radiotherapy	10 (83.3)
Mortality (n, %)	4 (33.3)

SD, standard deviation.

### 3.4 Adjuvant therapy

In total, the histological results indicated a malignant tumor entity in 9 (75%) patients and a benign tumor entity in 3 (25%) patients. All patients with malignant tumors underwent adjuvant treatment. Specifically, 77.8% (7 out of 9) received both chemotherapy and radiation. In contrast, for 22.2% (2 out of 9) patients with Clivus chordoma, proton radiation therapy alone was administered. Among patients with a benign tumor entity, 33.3% (1 out of 3) received adjuvant treatment. Notably, one patient diagnosed with a WHO Grade 1 meningioma, subsequently received adjuvant proton radiation therapy to target residual tumor remnants in cavernous sinus.

### 3.5 Systematic review

A systematic search of the literature yielded 881 papers. Following the exclusion of studies that did not report on pediatric cases, those with inadequate data, as well as studies that comprised over 5% of cases involving optic gliomas, pituitary adenomas, congenital anomalies like basal encephaloceles, cases of bone fibrous dysplasia, or those focusing solely on a single type of tumor such as meningiomas, a total of six papers were deemed suitable for inclusion. These studies encompassed a collective sample of 187 pediatric patients (Table 4).

**Table 4:** Late Effects Severity Score (LESS) scale^a^.

Category	1 Point	2 Points
neurology	1 isolated neurological late effect (mild ataxia, mild hemiparesis, cranial nerve palsy); pathological electroencephalography	any combination of >1 neurological late effect; seizures
endocrine	unsubstituted hormone deficit	≥1 hormone deficit
visual/auditory	uni- or bilat hearing impairment not requiring aid &/or visual impairment	unilat deafness or any hearing impairment requiring aid &/or visual impairment requiring special training/equipment
others	any treatment-related medical problem not listed, but not requiring medical intervention	any treatment-related medical problem not listed, but requiring medical intervention (such as a VP shunt)

a VP = ventriculoperitoneal.

The average age within this group was 10.9 years, with a male predominance of 56.7% and a female representation of 43.3%. With respect to the histological findings, 21 tumors were identified as malignant, constituting 36.2% of the cases, while the remaining 37 tumors, accounting for 63.8%, were benign. Due to the lack of detailed data differentiating benign from malignant tumors, the data from Brockmeyer and colleagues were omitted from this analysis. Complete tumor resection was successfully performed in 55.5% of the cases involving children. Radiation therapy was administered to 37.1% of the patients, whereas chemotherapy was given to 11.2%. There was a recurrence rate of 39.0% among the pediatric patients. The incidence of cerebrospinal fluid leakage postoperatively was recorded at 9.8%, and surgical infections were noted in 8.7% of the cases. According to the findings of this review, the mortality rate, because of the tumor progression or surgical complications, stood at 18.9%. These results are concisely presented in Table 5.

**Table 5:** Literatur review

Study	Number of Patients	Mean age (yrs)	Gender (male/female)	Tumor type	Extent of resection	Long-term impairment
Teo et al., 1999	26	10.5	18/8	26.9% Schwannoma/7.7% (each) chordoma, fibrous dysplasia, ependymoma, plexiform neurofibroma, ENB/3.9% (each) CP and sarcoma/26.8% Others	92.3% GTR/7.7% STR	69.2% None/19.2% deaths/15.4% facial weakness/11.5% deafness/7.7% dysphagia/7.7% blindness/15.4% others
Brockmeyer et al., 2003	55	9.8	30/25	23.6% Astrocytoma/10.9% Craniopharyngeoma/10.9 juvenil angiofibroma/5.5% MGM/3.6% Neurofibroma/45.5% Others	N/A	87.2% None/7.3% cranial nerve palsie/5.5% hemiparesis/
Hanbali et al., 2004	24	13.9	13/11	16.7% JNA/12.5% (each) RMS, schwanomma and sarcoma/8.3% desmoid tumor/4.2% chordoma/45.8% others	75.0% GTR/5.0% STR/4.2% biopsy	79.2% None/12.5% death/4.2% (each) hearing loss, hypoglossal neuropathy, facial and maxillary neuropathies, inadequate zygomatic growth
Mandonnet et al., 2008	42	13.5	21/21	14.3% Sarcoma/11.9% JNA/9.5% RMS/7.1% lymphangioma/7.1% chordoma/4.8% MGM/45.4% others	78.5% GTR/21.5% STR	85.7% None/31.0% Dead/4.8% (each) Visual impairment, Third nerve palsy and trigeminal neuropathy
Hayhurst et al., 2013	23	6.8	13/19	17.7% RMS/13.0% MGM/13.0% NB/8.7% angiofibroma/8.7% dermoid/4.3% (each) JNA, chordoma and sarcoma/26.0% others	52.2% GTR/43.5% STR/4.3% NTR	73.9% None/13.0% deaths/8.7% facial weakness
Ballestero et al., 2021	17	10.9	11/6	17.6% Schwannoma/5.9% Chondroid Chordoma/5.9% Mature teratoma/5.9% Epidermoid cyst/5.9% MGM/5.9 Rhabdomyosarcoma/5.9% Hemangiopericytoma/11.8% Sarcoma/5.9% JNA/29.3% Others	35.3% GTR/64.7% STR	23.5% None/29.4 facial paresis/29.4% hypoacusia/23.5% ocular motricity deficit/17.6% hydrocephalus 11.7% dysphonia/11.7% visual impairment/5.9% epilepsy/5.9% dysphagia
Overall	187	10.9	56.7%/43.3%		GTR 55.5 %	142/187 (76.0 %) None

M = male, F = female, CP = craniopharyngioma, EHE = epithelioid hemangioendothelioma, ENB = esthesioneuroblastoma, GTR = gross total resection, JVA = juvenile nasopharyngeal angiofibroma, MGM = meningioma, NB = neuroblastoma, N = number of patients, NI = no information, RMS = rhabdomyosarcoma, STR = subtotal resection.

### 3.6 Illustrative cases

#### 3.6.1 Case 1

**Fig. 1:**
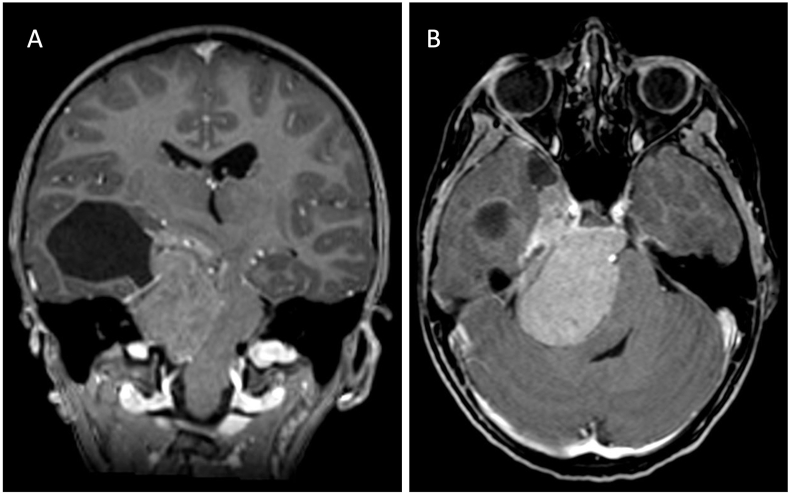
A – Coronar T1-weightet MRI with contrast agent showing right-sided petroclival contrast-enhancing mass with supra- and infratentorial tumor growth, compressing the brainstem and causing hydrocephalic changes. B – Axial T1-weighted MRI with contrast agent specifically demonstrates the compression of the fourth ventricle by the mass. The histological examination of the resected tissue revealed a meningeoma WHO Grade I.

A 9-year-old girl presented to our clinic on an emergency basis due to progressive headaches, gait instability, right-sided facial paralysis, and abducens nerve palsy. MRI imaging of the skull revealed a right-sided petroclival contrast-enhancing mass with supra- and infratentorial tumor growth, compressing the brainstem and causing hydrocephalic changes (4 × 4 cm). Initially, an external ventricular drain (EVD) was placed. The resection of the mass was performed in two stages. First, the resection of the infratentorial part was carried out through a right retrosigmoid approach. Due to strong adhesions of the tumor, thin sheet remnants near the basilar artery and in the area of the brainstem were left. The supratentorial parts were operated through a pterional approach. Tumor remnants had to be left due to adhesions in the area of the middle cerebral artery (M1) and near the internal carotid artery (ICA). Postoperatively, the patient exhibited a House-Brackmann grade 3 facial paralysis, which regressed postoperative to Grade II after 6 weeks then 1 year later was completely normal HB Grade I. The EVD was removed after a clamping trial, with no need for a shunt. The histological diagnosis revealed a Meningioma WHO Grade I. Following a decision by an interdisciplinary tumor board, adjuvant proton therapy with a total of 54 Gy was administered. Up to now (longest follow-up 48 months), no tumor progression has been observed (Fig. 1). Skull base meningiomas in children are most likely to be found in the anterior or middle fossa base, or involving the orbit and optic nerve sheath. Petroclival, suprasellar/parasellar, cerebellopontine angle, cavernous sinus, and foramen magnum tumors are very rare (Gump, 2015). The absence of female preponderance in pediatric meningiomas is reflected in the skull base subpopulation. Hence, we present this case of petroclival meningioma in a 9 years old girl.

3.6.2Case 2

**Fig. 2:**
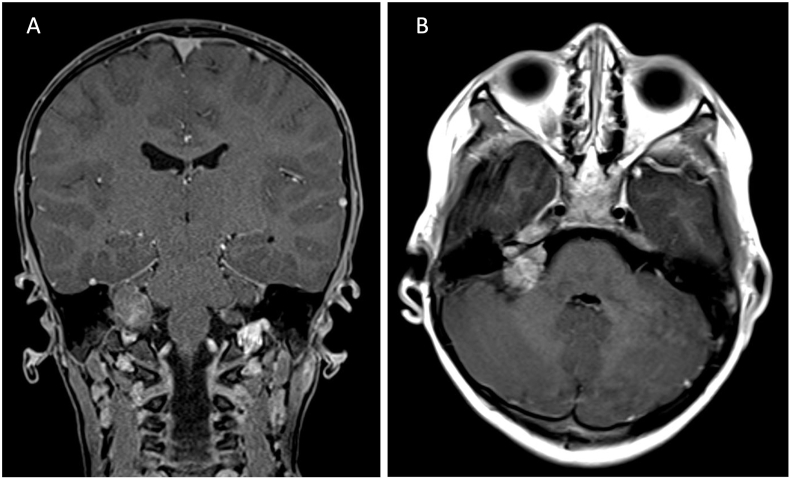
A – Coronar T1-weightet MRI with contrast agent showing a partially cystic, contrast-enhancing mass in the right cerebellopontine angle. B – Axial T1-weighted MRI with contrast agent specifically demonstrates the extension of the mass into the right internal acoustic meatus. The histological examination of the resected tissue revealed a atypic teratoidic rhabdoidic tumor (AT/RT) WHO Grade IV.

A 6-year-old boy presented clinically with right-sided House & Brackmann grade IV facial paralysis and hearing loss on the right. An MRI of the skull revealed a partially cystic, contrast-enhancing mass in the right cerebellopontine angle, extending into the internal auditory meatus, measuring 2 × 2 cm. The tumor resection was performed via a right retrosigmoid approach. Given the indistinct boundaries of the tumor in proximity to the facial nerve, decision was made to leave some residual tumor tissue to avoid potential injury of facial nerve. Postoperatively, the preexisting facial paralysis began to recede and resolved completely over 3 months. The histological diagnosis was an Atypical Teratoid/Rhabdoid Tumor (AT/RT) WHO Grade IV. An interdisciplinary tumor board recommended adjuvant radiotherapy and chemotherapy. The child succumbed to the malignancy of the tumor 24 months later (Fig. 2). ATRT are well known and reported in different locations of the CNS, but it is rare in the cerebellopontine angle cisterns, meninges, cranial nerves, spinal canal, and extradural sites (Zhao et al., 2015; Wykoff et al., 2008; Beschorner et al., 2006; Rorke et al., 1996). Hence, we demonstrate here this patient with ATRT in cerebellopontine angle mimicking vestibular schwannoma.

## 4 Discussion

Unfortunately, only few studies with a small patient population have examined outcomes, surgical approaches and long-term follow up in pediatric patients undergoing surgical treatment for skull base tumors. In the present retrospective single-institution case series study, twelve children with different tumor entities were analyzed. Notably, GTR and NTR were achieved in 58.3% of the patients. Postoperative complications were minimal, with hydrocephalus manifesting in two patients, a new postoperative neurological deficits occurred in one patient. Very rare tumor entities like Atypical Teratoid Rhabdoid Tumor (ATRT) as well as embryonal tumor with multilayered rosettes (ETMR) were also present along our collective.

Brockmeyer et al.‘s retrospective study analyzed 55 pediatric patients who underwent skull base surgery for a range of conditions, including tumors in 41 cases and fibrous dysplasia in two, alongside other issues like vascular lesions and epidural abscesses. The study reported encouraging outcomes, with a permanent neurological morbidity rate of 11%. Utilizing the Glasgow Outcome Scale (GOS), a remarkable 96% of these patients achieved scores of 4 or 5, indicative of favorable recoveries (Brockmeyer et al., 2003). In a similar study, Lang et al. examined a cohort of 20 children with diverse pathologies, such as juvenile nasopharyngeal angiofibroma, chordoma, schwannoma, and meningioma, all of whom underwent craniofacial access surgeries. Mirroring the findings of Brockmeyer et al. this group also exhibited positive postoperative outcomes, with 90% of the patients having successful recoveries. The GOS was again applied, revealing that while one patient was moderately disabled and another severely disabled, the majority were not significantly impaired. Notably, at their last follow-up, 18 of the 20 children were able to return to mainstream education, and only two required additional educational assistance due to pre-existing challenges (Lang et al., 1998). Contrastingly, Teo et al. reported on 26 children undergoing skull base surgeries for a variety of tumors, such as schwannomas, fibrous dysplasias, chordomas, and esthesioneuroblastomas. This study observed a considerable immediate postoperative complication rate of 57%, with long-term complications in 30.7% of the participants. Despite this, the study of Teo et al. highlighted a high rate of complete tumor resection at 92% and an 81% tumor-free survival rate after two years, showcasing a promising long-term outlook (Teo et al., 1999). In our study, we assessed the preoperative Lansky score, as an addition to the studies by Brockmeyer et al. Lang et al., and Teo et al. which did not report on preoperative score. In our study, the average preoperative Lansky score was 80. Postoperative similar to the studies by Brockmeyer et al. and Lang et al. our patients showed an improved postoperative Lansky score of 88.7. The LESS score in our study was 1. In contrast to the study by Teo et al. we observed a favorable outcome in the patients analyzed in our study, with a low rate of postoperative complications. Only one patient developed a new neurological deficit postoperatively.

In their examination of 24 children with skull base tumors, Hanbali et al. reported that 25% of the patients received either a subtotal resection or a biopsy during initial surgery. Postoperative complications were noted in 42% of the cases; however, the incidence of persistent neurological morbidity was substantially lower at 8%. The study highlighted an impressive 1-year and 5-year survival rate of 87%, although one patient did succumb to septicemia within 30 days post-surgery. The outcomes from Hanbali et al. underscore the potential benefits of aggressive resection in benign and low-grade malignant lesions, including chordomas, without the necessity for adjuvant therapy (Hanbali et al., 2004). In comparison to the study by Hanabali et al. a GTR or near NTR was achieved in 58.3% of the patients analyzed in our study. Furthermore, a postoperative complication was observed in one patient who developed a new neurological deficit, which resolved over time. In the study by Hanabali et al. the complication rate was 42%, with 8% of patients experiencing a persistent deficit. Two of our patients developed postoperative hydrocephalus, with one requiring a ventriculoperitoneal (VP) shunt and the other underwent endoscopic third ventriculostomy (ETV).

Hayhurst et al. offered insights into a diverse group of 23 pediatric patients, ranging from 13 months to 15 years, who underwent skull base surgeries for a variety of tumors, including meningiomas, schwannomas, rhabdomyosarcomas, neuroblastomas, angiofibromas, and chordomas. Over a median follow-up period of 60 months, a complete tumor resection was accomplished in just over half of the patients (52%), with the majority (57%) diagnosed with benign tumors. The typical length of hospitalization was approximately one week. Notably, there were three fatalities in the cohort: one during the perioperative phase and two resulting from tumor progression. Together, these studies reflect the complex landscape of pediatric skull base surgery, highlighting the delicate balance between achieving complete resection and minimizing postoperative complications and neurological morbidity. The collective findings underscore the importance of individualized treatment strategies tailored to the tumor type, location, and patient age, with an overarching goal of optimizing outcomes and quality of life for these young patients.

Our findings indicate the following distribution of tumor locations: 50% were located in the posterior cranial fossa, 33% in the middle cranial fossa, and another 17% in the anterior cranial base. Specifically, we observed that the most common tumor locations were the cerebellopontine angle and the clivus bone, accounting for 41.6% and 25% of cases respectively. Belestero et al. reported tumor localization mostly in the cerebellopontine angle was the primary tumor site in 23.5%, the sphenoid bone in 23.5%, and the petrous temporal bone in 17.6% (Ballestero et al., 2021). This distribution contrasts with the findings of Hayhurst et al. who reported that 17.4% of tumors were found extending to the paranasal and frontal areas, 13% each in the orbit and infratemporal area, and lower frequencies of 8.7% each in the cerebellopontine angle and sphenoid bone. The present study, however, could not perform a direct comparison of tumor locations with those in the studies analyzed in the review, due to the diverse nature of the data. This challenge stems from the absence of a standardized approach for categorizing lesion locations.

In our study, the preferred surgical methods were the retrosigmoid approach, followed by the pterional approach and an endoscopic endonasal and a transoral approach. This selection aligns with Teo et al. who reported using cranio-orbito-zygomatic approaches in 23.1% of their cases and retrosigmoid approaches in 19.2% (Teo et al., 1999). Conversely, Brockmeyer et al. predominantly used orbitozygomatic osteotomies (46.4%) and did not employ retrosigmoid approaches (Ballestero et al., 2021). However, due to varied methodologies across different studies, a direct comparison of surgical techniques was not feasible in this review. Children undergoing skull base surgery face unique anatomical challenges compared to adults. Key considerations include the ability to achieve hemostasis, which depends on the lesion's location and pathology, the size of the lesion, and the potential need for vascular reconstruction. Tackling complex tumors at the skull base often necessitates intricate surgical strategies, sometimes involving a combination of methods like a two staged method combining the retrosigmoidal approache and pterional approach for resecting tumors in the petroclival cavity with supratentorial extension. Implementing endoscopic techniques in the pediatric skull base lesions population, particularly in younger patients, presents unique challenges. These include the small size of nasal passages, developing air sinuses, and the potential impact on craniofacial growth. Factors such as the extent of sphenoid sinus pneumatization, the intercarotid distance, and the width of the piriform aperture must be carefully assessed. Additionally, repairing skull base defects in children can be problematic due to the larger size of endoscopes and surgical instruments relative to the patient's anatomy (Deopujari et al., 2019). Consequently, there is a critical need to innovate new methods for closure to mitigate the risk of cerebrospinal fluid (CSF) leaks (Shah et al., 2019). While endoscopic surgery for skull base tumors is a well-established practice in adult patients, its adaptation for pediatric cases has not seen the same level of development or acceptance (Kim et al., 2019).

Concerning the prevalence of CSF leakage following such approaches, Belestero et al. and Mandonnet et al. incorporated endoscopic assistance in one of their cases, although it's not specified if a CSF leak occurred (Ballestero et al., 2021; Mandonnet et al., 2008). In contrast, Teo et al. employed a fully endoscopic method in two instances without any CSF leaks and provided endoscopic assistance in another case, which did result in a CSF leak (Teo et al., 1999). Stapleton et al. focusing on pediatric skull base tumor surgeries using full endoscopic endonasal techniques, reported postoperative CSF leaks in 23% of their cases (11 out of 47) (Stapleton et al., 2017). These cases necessitated further surgical intervention for repair, but it's important to note that this study only included patients who exhibited CSF leaks intraoperatively. Along our cohort, two patients underwent fully endoscopic surgery for the resection of clivus chordomas. In another case, an endoscopic-assisted approach was adopted for the resection of a suprasellar epidermoid cyst, aimed at achieving better control in the region of the optic system. No CSF leak was observed.

While surgical techniques for addressing skull base tumors are often developed with adults in mind, these approaches can also be applied to pediatric patients. However, the distinct anatomical features of children, including smaller size and ongoing development of the skull, pose specific challenges for pediatric neurosurgeons (Tsai et al., 2002). The decision to undertake skull base surgery in children requires careful consideration, weighing the therapeutic benefits against the potential long-term effects on craniofacial growth, donor site morbidity, and psychosocial implications. There are notable anatomical differences between children and adults in terms of skull base morphology. For instance, children have a smaller cranial base and maxillofacial complex, thinner cranial bones, and possibly flatter floors of the frontal and middle cranial fossae (Brockmeyer et al., 2003). Despite these differences, common skull base surgical approaches, when suitably modified, can be effectively and safely used in the pediatric population. Key surgical considerations include accommodating the smaller, thinner skull in procedures like pinning, drilling, handling, and plating (LoPresti et al., 2018).

## 6 Conclusion

Employing a comprehensive, interdisciplinary strategy, pediatric patients generally endure skull base surgeries effectively, exhibiting a minimal incidence of enduring neurological complications and negligible long-term effects. The treatment of skull base tumors in children, given their rarity, should be conducted in specialized centers due to the higher expertise of all involved disciplines. Future perspectives should focus on developing robust protocols and guidelines which may be enabled via collection of these data in a prospective multicentric registry fashion. Also, there is a need for future prospective research that includes neuropsychological evaluations and assessments of quality of life to thoroughly understand the impact of both malignant and benign skull base tumors on the development of children.

## 5 Limitations

This retrospective study is subject to certain limitations arising from incomplete documentation, which in turn posed challenges when conducting a thorough analysis. Notably, the relatively small sample size of 12 patients may restrict the statistical significance of the obtained results. To address these limitations, it is recommended to undertake future studies encompassing larger sample sizes and employing a prospective design. It is crucial to administer standardized neurologic, ophthalmological, and endocrinological assessments at standardized time intervals. Furthermore, conducting a multicenter study involving various hospitals and comparing both transcranial and transnasal approaches would yield more robust and generalizable findings. The robustness of this study is notably underscored by the comprehensive objectification of functional outcomes via the LESS and Lansky Performance Scores. Additionally, this analysis encompasses a diverse array of skull base surgical approaches, including transoral, endoscopic endonasal, and endoscopically assisted techniques. Moreover, the inclusion of rare tumor types, such as Atypical Teratoid/Rhabdoid Tumors (ATRT) and Embryonal Tumors with Multilayered Rosettes (ETMR), further enhances the study's significance.
